# Rescue therapy after failed thrombectomy in medium/distal vessel occlusions: A retrospective analysis of an international, multi-center registry

**DOI:** 10.1177/23969873241311152

**Published:** 2025-01-04

**Authors:** Aikaterini Anastasiou, Alex Brehm, Tomas Dobrocky, Adnan Mujanovic, Marta de Dios Lascuevas, Tomas Carmona Fuentes, Alfonso López-Frías López-Jurado, Blanca Hidalgo Valverde, Ansgar Berlis, Christoph J. Maurer, Thanh N Nguyen, Mohamad Abdalkader, Piers Klein, Guillaume Thevoz, Patrik Michel, Marius Kaschner, Daniel Weiss, Andrea M. Alexandre, Alessandro Pedicelli, Paolo Machi, Gianmarco Bernava, Shuntaro Kuwahara, Kazutaka Uchida, Jason Wenderoth, Anirudh Joshi, Grzegorz Karwacki, Lehel-Barna Lakatos, Agostino Tessitore, Sergio Lucio Vinci, Amedeo Cervo, Claudia Rollo, Ferdinand Hui, Aaisha Siddiqua Mozumder, Daniele Giuseppe Romano, Gianmarco Flora, Nitin Goyal, Vivek Batra, Violiza Inoa, Christophe Cognard, Matúš Hoferica, Riitta Rautio, Daniel Kaiser, Hanna Alph, Julian Clarke, Nick Hug, Alma Koch, Victor Schulze-Zachau, Nikki Rommers, Mira Katan, Marios-Nikos Psychogios

**Affiliations:** 1 Department of Neuroradiology, University Hospital Basel, Basel, Switzerland; 2 Department of Diagnostic and Interventional Neuroradiology, Inselspital University Hospital Bern, University of Bern, Bern, Switzerland; 3 Interventional Neuroradiology, Vall d’Hebron University Hospital, Barcelona, Spain; 4 Interventional Neuroradiology, Hospital Clínico San Carlos, Madrid, Spain; 5 Stroke Unit, Department of Neurology, Hospital Clínico San Carlos, Madrid, Spain; 6 Diagnostic and Interventional Neuroradiology, University Hospital Augsburg, Augsburg, Germany; 7 Department of Radiology, Boston Medical Center, Boston University Chobanian & Avedisian School of Medicine, Boston, MA, USA; 8 Stroke Center, Neurology Service, Department of Clinical Neurosciences, Lausanne University Hospital, Lausanne, Switzerland; 9 Medical Faculty, Department of Diagnostic and Interventional Radiology, University Duesseldorf, Düsseldorf, Germany; 10 UOSA Neuroradiologia Interventistica, Fondazione Policlinico Universitario A. Gemelli IRCCS, Roma, Italy; 11 Division of Neuroradiology, Geneva University Hospitals, Geneva, Switzerland; 12 Department of Neurosurgery, Hyogo Medical University, Nishinomiya, Japan; 13 Institute of Neurological Sciences, Prince of Wales Hospital, Randwick, NSW, Australia; 14 Prince of Wales Clinical School, University of New South Wales, Sydney, Australia; 15 Department of Radiology and Nuclear Medicine, Cantonal Hospital Lucerne, Lucerne, Switzerland; 16 Department of Neurology and Neurorehabilitation, Kantonsspital Lucerne, Lucerne, Switzerland; 17 Neuroradiology Unit, University Hospital A.O.U. “G. Martino” - Messina, Messina, Italy; 18 Department of Biomedical, Dental and Morphological and Functional Imaging (BIOMORF), University of Messina, Messina, Italy; 19 Department of Neuroradiology, ASST Grande Ospedale Metropolitano Niguarda (Niguarda Ca’ Granda), Milan, Italy; 20 Neuroscience Institute, The Queen’s Medical Center, Honolulu, HI, USA; 21 University of Hawaii, Honolulu, HI, USA; 22 Unit of Interventional Neuroradiology, University Hospital AOU Salerno, Italy; 23 Department of Neurology, University of Tennessee Health Science Center, Memphis, TN, USA; 24 Department of Neurological Surgery, Semmes-Murphey Clinic, Memphis, TN, USA; 25 Neuroradiology Department, Toulouse University Hospital, INSERM, U1048 and Université Toulouse 3, I2MC, Toulouse, France; 26 Department of Radiology, Turku University Hospital, Turku, Finland; 27 Institute of Neuroradiology, University Hospital Carl Gustav Carus, Technische Universität Dresden, Dresden, Germany; 28 Department of Neurological Surgery, University of Washington, Seattle, WA, USA; 29 Department of Clinical Research, University of Basel, University Hospital Basel, Basel, Switzerland; 30 Department of Neurology, Stroke Center, University and University Hospital of Basel, Switzerland

**Keywords:** Acute ischemic strokes, endovascular treatment, rescue stenting, intracranial atherosclerotic disease

## Abstract

**Background:**

There are limited therapeutic options in cases of failed reperfusion (modified thrombolysis in cerebral infarction [mTICI] score < 2b) after stent-retriever and/or aspiration based endovascular treatment (EVT) for acute ischemic stroke. Despite the absence of data supporting its use, rescue therapy (balloon angioplasty and/or stent implantation) is often utilized in such cases. Studies are limited to large vessel occlusions, while the outcomes and complications after rescue therapy in medium/distal vessel occlusions (MDVOs) have not been reported. This study aims to report the outcomes of rescue therapy in MDVO stroke patients.

**Methods:**

We performed an analysis of the “Blood pressure and Antiplatelet medication management after reScue angioplasty after failed Endovascular treatment in Large and distal vessel occlusions with probable IntraCranial Atherosclerotic Disease” (BASEL ICAD) retrospective registry. All MDVO stroke patients were included in the analysis.

**Results:**

Out of the 718 registry patients, 87 (12.1%) presented with an MDVO. Fifty-six patients (64.4%) showed an occlusion of the M2 segment of the middle cerebral artery. Rescue stenting was performed in 78 patients (89.7%) while balloon angioplasty alone was performed in 9 patients (10.3%). Successful reperfusion (mTICI score ⩾ 2b) was achieved in 73 (83.9%) patients after rescue therapy. Symptomatic intracranial hemorrhage (sICH) occurred in 8 patients (9.2%) and post-treatment stent occlusion in 12 patients (13.8%). Ninety days mortality was 20.7%. Twenty-eight patients (32.2%) achieved functional independence at 90 days (modified Rankin Scale 0–2).

**Conclusion:**

Rescue therapy with stenting and/or balloon angioplasty in patients undergoing EVT for isolated MDVO with suspected underlying intracranial atherosclerotic disease is an effective reperfusion strategy but is associated with complications and poor functional outcomes.

## Graphical abstract

**Graphical abstract:**
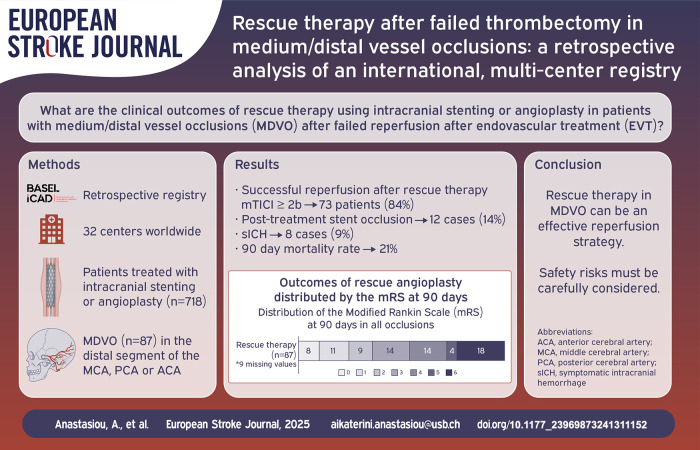

## Introduction

Acute ischemic stroke (AIS) is a major contributor to mortality and disability, ranking third in terms of loss of quality-adjusted life years.^1,2^ For patients experiencing AIS due to a large vessel occlusion (LVO), endovascular treatment (EVT) has emerged as a standard treatment.^3^ Over recent years and due to growing confidence with the procedure, EVT is often also extended to patients with medium/distal vessel occlusions (MDVOs), constituting 20%–40% of all AIS cases.^4–7^ Rapid and complete reperfusion achieved after EVT remains the primary predictor of positive outcomes.^8^ Failed reperfusion (mTICI score < 2b) occurs in 10%–20% of cases, primarily due to underlying intracranial atherosclerotic disease (ICAD), which results in vessel stenosis or in-situ thrombosis leading to instant re-occlusion.^9,10^ It is often observed that after initial successful reperfusion, the artery reoccludes within the time span of minutes. Failure to achieve reperfusion is strongly associated with worse clinical outcomes, with rates of severe disability and death surpassing 70%.^11^ There is no consensus on the best approach to handle failed reperfusion after EVT.^12^

**Figure 1.**
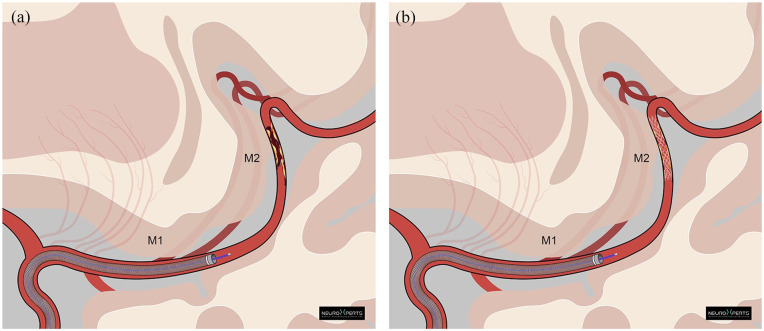
Rescue stenting paradigm of an ICAD related occlusion in the M2 Segment of the MCA.

Rescue therapy with intracranial stenting or angioplasty has emerged as a potential treatment in such cases. Often, AIS from ICAD is caused by thrombosis from an activated atherosclerotic plaque. Thus, even with conventional removal of the thrombus from the occluded site, an activated plaque remains, and re-occlusion can occur. The local recurring thrombus can be potentially treated with the implantation of a stent or with balloon angioplasty alone.^13,14^ (Figure 1) However, this approach has not been evaluated in a large cohort of MDVOs with failed reperfusion. Furthermore, EVT in MDVO is currently under investigation in multiple randomized controlled trials and a matter of debate.^15^

In this study we report the outcomes of patients undergoing rescue therapy during the EVT for the treatment of AIS due to MDVO.

## Methods

We performed a retrospective analysis of the “Blood pressure and Antiplatelet medication management after reScue angioplasty after failed Endovascular treatment in Large and distal vessel occlusions with probable IntraCranial Atherosclerotic Disease” (BASEL ICAD) retrospective registry. Data were obtained from 32 EVT centers. Adult patients (Age ⩾ 18 years) were included in the BASEL ICAD registry if they underwent rescue therapy for the treatment of an LVO or MDVO between January 1st, 2019, and December 31st, 2023. Data from participating centers were curated by reviewing patient charts and procedure notes from the electronic medical records. This registry was approved by the applicable ethics committee (BASEC ID 2024-00904) with a waiver of consent.

For the current analysis only patients who had an isolated primary MDVO were included. We defined MDVO as an occlusion of the co- or non-dominant M2, the M3 or M4 segment of the middle cerebral artery (MCA), the A1, A2 or A3 segment of the anterior cerebral artery (ACA) or the P1, P2 or P3 segment of the posterior cerebral artery (PCA).

The primary clinical outcome was the modified Rankin Scale (mRS) at 90 days, while the primary procedural outcome was the rate of successful reperfusion (defined as a modified thrombolysis in cerebral infarction [mTICI] score of ⩾ 2b) at the end of the procedure. Secondary outcomes of interest included the National Institute of Health Stroke Scale (NIHSS) at 24 h, symptomatic intracranial hemorrhage (sICH) at 24 h scored with the Heidelberg Bleeding Classification, post-treatment stent occlusion (within 24 h and after day 7) and procedural complications. (Table 1) In addition, we performed a matching of MDVO with LVO patients from the BASEL-ICAD registry with ratio 1:2 using age, NIHSS at admission, pre-stroke mRS, intravenous thrombolysis (IVT), and stenting (vs balloon angioplasty alone) as matching variables. (Supplemental Table 2).

**Table 1.** Patient characteristics and outcomes.

Characteristics and outcomes	Overall	M2	M3/M4, ACA, PCA	Missing (%)
Number	87	56	31	
Age	71.6 (12.8)	70.1 (13.6)	74.2 (11.1)	0.0
Sex				0.0
Female	36 (41.4)	28 (50.0)	8 (25.8)	
Male	51 (58.6)	28 (50.0)	23 (74.2)	
Race				12.6
African American	8 (10.5)	7 (14.9)	1 (3.4)	
Asian	5 (6.6)	4 (8.5)	1 (3.4)	
White	63 (82.9)	36 (76.6)	27 (93.1)	
*Missing*	*11 (12.6)*	*9 (16.1)*	*2 (6.5)*	
Hypertension	66 (75.9)	44 (78.6)	22 (71.0)	2.3
Dyslipidemia	29 (33.3)	17 (30.4)	12 (38.7)	18.4
Diabetes mellitus	20 (23.0)	15 (26.8)	5 (16.1)	2.3
Coronary artery occlusive disease	12 (13.8)	8 (14.3)	4 (12.9)	2.3
Current or past smoking	20 (23.0)	16 (28.6)	4 (12.9)	3.4
Atrial fibrillation	9 (10.3)	6 (10.7)	3 (9.7)	2.3
History of stroke or TIA	21 (24.1)	14 (25.0)	7 (22.6)	2.3
Pre-stroke mRS				1.1
0	51 (58.6)	33 (58.9)	18 (58.1)	
1	15 (17.2)	11 (19.6)	4 (12.9)	
2	9 (10.3)	6 (10.7)	3 (9.7)	
3	9 (10.3)	4 (7.1)	5 (16.1)	
4	2 (2.3)	1 (1.8)	1 (3.2)	
*Missing*	1 (1.1)	1 (1.8)	0 (0.0)	
Previous anticoagulation use	7 (8.0)	6 (10.7)	1 (3.2)	10.3
Previous antiplatelet use				9.2
No	59 (67.8)	37 (66.1)	22 (71.0)	
Single	18 (20.7)	11 (19.6)	7 (22.6)	
Double	2 (2.3)	2 (3.6)	0 (0.0)	
*Missing*	8 (9.2)	6 (10.7)	2 (6.5)	
NIHSS admission	9.0 [5.5, 14.5]	10.0 [5.8, 14.2]	8.0 [5.5, 14.5]	0.0
IVT	24 (27.6)	10 (17.9)	14 (45.2)	0.0
Time onset to groin puncture (min)	428.0 [204.0, 770.5]	450.0 [212.5, 735.8]	428.0 [203.0, 780.0]	13.8
Occluded vessel				0.0
A1/A2	7 (8.0)	0 (0.0)	7 (22.6)	
A3/A4	2 (2.3)	0 (0.0)	2 (6.5)	
M2	56 (64.4)	56 (100.0)	0 (0.0)	
M3/M4	1 (1.1)	0 (0.0)	1 (3.2)	
P1	17 (19.5)	0 (0.0)	17 (54.8)	
P2/P3	4 (4.6)	0 (0.0)	4 (12.9)	
Occlusion side				2.3
Left	54 (62.1)	35 (62.5)	19 (61.3)	
Right	31 (35.6)	21 (37.5)	10 (32.3)	
*Missing*	2 (2.3)	0 (0.0)	2 (6.5)	
Etiology rescue stenting				9.2
ICAD	65 (74.7)	41 (73.2)	24 (77.4)	
Dissection	9 (10.3)	6 (10.7)	3 (9.7)	
Hard thrombus	5 (5.7)	3 (5.4)	2 (6.5)	
*Missing*	8 (9.2)	6 (10.7)	2 (6.5)	
Outcomes				
mRS 90 days				10.3
0	8 (9.2)	7 (12.5)	1 (3.2)	
1	11 (12.6)	9 (16.1)	2 (6.5)	
2	9 (10.3)	7 (12.5)	2 (6.5)	
3	14 (16.1)	12 (21.4)	2 (6.5)	
4	14 (16.1)	8 (14.3)	6 (19.4)	
5	4 (4.6)	3 (5.4)	1 (3.2)	
6	18 (20.7)	7 (12.5)	11 (35.5)	
*Missing*	9 (10.3)	3 (5.4)	6 (19.4)	
Functional independence at 90 days	28 (32.2)	23 (41.1)	5 (16.1)	10.3
sICH	8 (9.2)	4 (7.1)	4 (12.9)	4.6
NIHSS 24 h	9.0 [4.0, 17.0]	8.0 [4.0, 15.5]	13.0 [4.0, 18.0]	11.5
Death	18 (20.7)	7 (12.5)	11 (35.5)	10.3

## Results

Eighty-seven (12.1%) out of 718 BASEL ICAD registry patients had an MDVO. Seventy-eight (89.7%) underwent intracranial stenting, and nine (10.3%) angioplasty alone. The mean age was 71.6 years (±12.8) and 41.4% were female. Seventy-five patients (86.1%) had a pre-stroke mRS ⩽ 2 (51 of whom were mRS 0) and 11 (12.6%) > 2 (pre-stroke mRS was missing in one patient). The median admission NIHSS was 9 (Interquartile-Range [IQR] 5.5–14.5). Twenty-four patients (27.6%) received IVT. Median time of onset to groin puncture was 428 [IQR 204–770.5] minutes and median time of onset to recanalization was 508 min [IQR 308–800]. The most prevalent occlusion location was the M2 segment (56 patients, 64.4%). Other occlusion locations were the A1/A2 (7 patients, 8%), A3/A4 (2 patients, 2.3%), M3/M4 (1 patients, 1.1%), P1 (17 patients, 19.5%) and the P2/P3 segments (4 patients, 4.6%). (see Table 1 for baseline characteristics).

**Figure 2.**
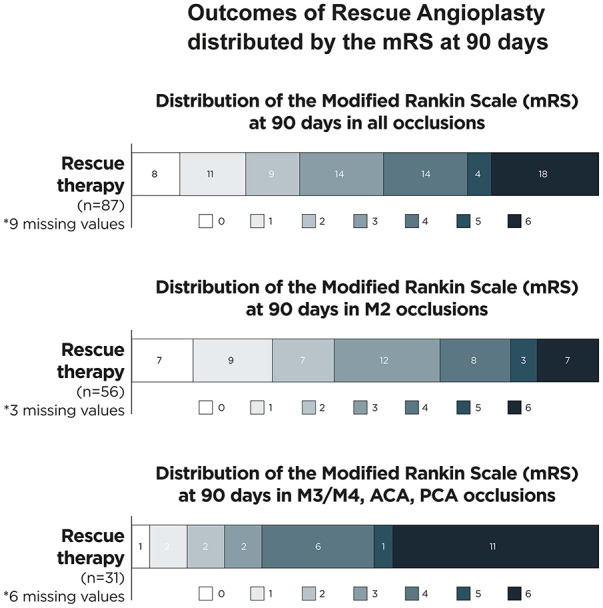
Distribution of the modified rankin scale (mRS) at 90 days in different distal vessel locations.

The mRS at 90 days was 0 in 8 cases (9.2%), 1 in 11 cases (12.6%), 2 in 9 cases (10.3%), 3 in 14 cases (16.1%), 4 in 14 cases (16.1%), 5 in 4 cases (4.6%), 6 in 18 cases (20.7%). We had 9 missing values. (Figure 2). Twenty-eight patients (32.2%) achieved a good functional outcome (defined as mRS 0–2) at 90 days. Median NIHSS at 24 h was 9 [IQR 4–17]. The highest mTICI score achieved prior to rescue therapy was 2a or lower in 48 patients (55.1%). The AIS etiology was deemed from the treating physician to be ICAD in 74.7% of the patients (*n* = 65). After rescue therapy mTICI ⩾ 2b was achieved in 73 (83.9%) patients, while 49 (56.3%) had mTICI ⩾ 2c. Rescue therapy was performed after a median of 2 (IQR 1–3) EVT passes. (see Table 2 for intervention characteristics). The most used stents were the CREDO^®^ stent (Acandis, Pforzheim, Germany) in 27 patients, the Neuroform Atlas (Stryker, Kalamazoo, US) in 9 patients and the Acclino^®^ stent (Acandis, Pforzheim, Germany) in 8 patients. Eighty-three (94.3%) patients received at least one peri-procedural antiplatelet medication: A single agent in 41 (47.1%), two agents in 33 (37.9%) and three agents in 9 (10.3%). (Supplemental Table 1). Efficacy and safety outcomes were not different between patients receiving IVT or no IVT.

**Table 2.** Intervention.

Characteristics	Overall	M2	M3/M4, ACA, PCA	Missing (%)
Number	87	56	31	
Anesthesia				6.9
General	42 (48.3)	25 (44.6)	17 (54.8)	
Sedation	39 (44.8)	26 (46.4)	13 (41.9)	
*Missing*	6 (6.9)	5 (8.9)	1 (3.2)	
Access site				6.9
Femoral	75 (86.2)	49 (87.5)	26 (83.9)	
Radial	6 (6.9)	2 (3.6)	4 (12.9)	
*Missing*	6 (6.9)	5 (8.9)	1 (3.2)	
Highest mTICI achieved prior to rescue therapy				6.9
0	27 (31.0)	20 (35.7)	7 (22.6)	
1	13 (14.9)	8 (14.3)	5 (16.1)	
2a	8 (9.2)	4 (7.1)	4 (12.9)	
2b	16 (18.4)	9 (16.1)	7 (22.6)	
2c	8 (9.2)	6 (10.7)	2 (6.5)	
3	9 (10.3)	5 (8.9)	4 (12.9)	
*Missing*	6 (6.9)	4 (7.1)	2 (6.5)	
mTICI after rescue therapy				1.1
0	9 (10.3)	5 (8.9)	4 (12.9)	
1	2 (2.3)	1 (1.8)	1 (3.2)	
2a	2 (2.3)	1 (1.8)	1 (3.2)	
2b	24 (27.6)	16 (28.6)	8 (25.8)	
2c	15 (17.2)	9 (16.1)	6 (19.4)	
3	34 (39.1)	24 (42.9)	10 (32.3)	
*Missing*	1 (1.1)	0 (0.0)	1 (3.2)	
Number of passes	2.0 [1.0, 3.0]	2.0 [1.0, 4.0]	2.0 [2.0, 3.0]	2.3
Stenting	78 (89.7)	49 (87.5)	29 (93.5)	0.0
Balloon angioplasty				0.0
No	32 (36.8)	23 (41.1)	9 (29.0)	
Prestent	35 (40.2)	22 (39.3)	13 (41.9)	
Poststent	8 (9.2)	2 (3.6)	6 (19.4)	
Both	3 (3.4)	2 (3.6)	1 (3.2)	
Alone	9 (10.3)	7 (12.5)	2 (6.5)	
Time onset to recanalization (min)	508.0 [308.0, 800.0]	475.5 [321.5, 763.5]	515.0 [270.0, 830.0]	20.7
Complications				0.0
No	65 (74.7)	41 (73.2)	24 (77.4)	
SAH/vessel perforation	11 (12.6)	8 (14.3)	3 (9.7)	
Dissection	3 (3.4)	2 (3.6)	1 (3.2)	
Femoral/retroperitoneal hematoma	3 (3.4)	2 (3.6)	1 (3.2)	
Other	5 (5.7)	3 (5.4)	2 (6.5)	
Post-treatment stent occlusion	12 (13.8)	3 (5.4)	9 (29.0)	9.2
Timing post-treatment stent occlusion				89.7
Within 24h	7 (8.0)	3 (5.4)	4 (12.9)	
After day 7	1 (1.1)		1 (3.2)	
*Missing/Not applicable*	79 (90.9)	53 (94.6)	26 (83.9)	
Periprocedural antiplatelets	82 (94.3)	51 (91.1)	31 (100.0)	0.0
Number of periprocedural antiplatelets				4.6
1	41 (47.1)	25 (44.6)	16 (51.6)	
2	33 (37.9)	19 (33.9)	14 (45.2)	
3	9 (10.3)	8 (14.3)	1 (3.2)	
*Missing/Not applicable*	4 (4.6)	4 (7.1)	0 (0.0)	
Postprocedural antiplatelets	81 (93.1)	52 (92.9)	29 (93.5)	1.1
Number of periprocedural antiplatelets				5.7
1	14 (16.1)	9 (16.1)	5 (16.1)	
2	68 (78.2)	44 (78.6)	24 (77.4)	
*Missing/Not applicable*	5 (5.7)	3 (5.4)	2 (6.5)	

In 11 cases (12.6%) intraprocedural subarachnoid hemorrhage (SAH) or vessel perforation occurred. Post- treatment stent occlusion was reported in 12 (13.8%) patients and 8 patients (9.2%) had sICH. The risk of occlusion was higher in patients with postinterventional treatment with a single antiplatelet agent. The 90 day mortality rate was 20.7%. In the matched comparison with the LVO patients form the BASEL-ICAD registry, no significant differences were found. (Supplemental Table 2).

## Discussion

The use of EVT is increasingly common in MDVO patients based on extrapolation of strong evidence in the treatment of LVO. This extrapolation extends to the potential use of rescue therapy in such patients. This is, to the best of our knowledge, the first study of rescue therapy in a large cohort of MDVO AIS patients. While it must be taken into considerations that comparisons between studies are to be interpreted with caution due to possible unidentified factors, it appears that outcomes in MDVO patients requiring intracranial stenting or angioplasty are worse compared to the overall population of MDVO patients undergoing EVT.^16–18^

Good functional outcome was achieved in 32.2% of the patients in the current study. Other studies examining EVT for MDVO patients (with or without angioplasty) have consistently reported higher rates of good functional outcome.^16,19,20^ In the INTERRSeCT/PRoveIT study the rate of mRS 0–2 was 67.4%.^16^ The analysis further indicated that excellent outcome (mRS 0–1) was associated with successful early reperfusion. In the HERMES meta-analysis on M2 patients, the rate of good functional outcome was 58.2% in patients undergoing EVT.^20^

This is despite, the fact that these patients presented with a more severe stroke than those in the current report, and successful reperfusion rates were lower in the HERMES meta-analysis (59.2% in HERMES vs 84.8% in the current report).

Rates of sICH were similar between our study and the INTERRSeCT/PRoveIT registry, while in the HERMES meta-analysis, 0% of sICH was reported. Mortality at 90 days was higher in our study (20.7%) compared to 8.9% in the INTERSECT/PROACT registry and 11.9% in the HERMES meta-analysis.

We hypothesize that the difference in good outcomes seen in EVT for MDVO with rescue therapy are due to one or more of the following: The fact that in our study only MDVOs were included, with possible technical and safety considerations arising from the small vessel size. The overall higher number of passes (since we include a cohort of failed thrombectomies), a higher rate of ischemic or hemorrhagic complications after rescue therapy compared to conventional EVT, and the need for antiplatelet medication to prevent vessel reocclusion. Even with such medication, stent-reocclusion occurred in 13.8% of patients.

## Limitations

The limitations of this study are similar to all retrospective studies and mainly stem from the uncontrolled inclusion of patients (i.e. selection bias). A further limitation is the considerable variability in the antiplatelet treatment regimens and their unknown impact on patient outcome. Finally, because no core lab was available, radiological and clinical results were self-adjudicated. The sample size is limited. The most common occlusion site was the non-dominant M2-segment of the middle cerebral artery. The other locations are underrepresented. However, given that we could analyze a large number of patients for a relatively rare procedure, and given the multicenter nature of the registry, this study may meaningfully impact future trials of rescue therapy for MDVO.

## Conclusion

Rescue therapy with stenting and/or balloon angioplasty in patients undergoing EVT for isolated MDVO with suspected underlying ICAD is an effective reperfusion strategy but is associated with complications and poor functional outcomes.

## Supplemental Material

sj-docx-1-eso-10.1177_23969873241311152 – Supplemental material for Rescue therapy after failed thrombectomy in medium/distal vessel occlusions: A retrospective analysis of an international, multi-center registry Supplemental material, sj-docx-1-eso-10.1177_23969873241311152 for Rescue therapy after failed thrombectomy in medium/distal vessel occlusions: A retrospective analysis of an international, multi-center registry by Aikaterini Anastasiou, Alex Brehm, Tomas Dobrocky, Adnan Mujanovic, Marta de Dios Lascuevas, Tomas Carmona Fuentes, Alfonso López-Frías López-Jurado, Blanca Hidalgo Valverde, Ansgar Berlis, Christoph J. Maurer, Thanh N Nguyen, Mohamad Abdalkader, Piers Klein, Guillaume Thevoz, Patrik Michel, Marius Kaschner, Daniel Weiss, Andrea M. Alexandre, Alessandro Pedicelli, Paolo Machi, Gianmarco Bernava, Shuntaro Kuwahara, Kazutaka Uchida, Jason Wenderoth, Anirudh Joshi, Grzegorz Karwacki, Lehel-Barna Lakatos, Agostino Tessitore, Sergio Lucio Vinci, Amedeo Cervo, Claudia Rollo, Ferdinand Hui, Aaisha Siddiqua Mozumder, Daniele Giuseppe Romano, Gianmarco Flora, Nitin Goyal, Vivek Batra, Violiza Inoa, Christophe Cognard, Matúš Hoferica, Riitta Rautio, Daniel Kaiser, Hanna Alph, Julian Clarke, Nick Hug, Alma Koch, Victor Schulze-Zachau, Nikki Rommers, Mira Katan and Marios-Nikos Psychogios in European Stroke Journal
