# Supplementary material for: Rescue therapy after failed thrombectomy in medium/distal vessel occlusions: A retrospective analysis of an international, multi-center registry
Source: Eur Stroke J. 2025 Jan 4;10(3):713–20. doi: 10.1177/23969873241311152 (PMC11700384; doi:10.1177/23969873241311152)
Supplement: sj-docx-1-eso-10.1177_23969873241311152 – Supplemental material for Rescue therapy after failed thrombectomy in medium/distal vessel occlusions: A retrospective analysis of an international, multi-center registry [file sj-docx-1-eso-10.1177_23969873241311152.docx]

| **Supplement Table 1: Antiplatelet Management** | | | | |
| --- | --- | --- | --- | --- |
|  | **Overall** | **M2** | **M3/M4, ACA, PCA** | **Missing (%)** |
| n | 88 | 57 | 31 |  |
| Heparin (I.U.) | 1641.5 (2329.5) | 1486.5 (2218.9) | 2000.0 (2607.7) | 39.1 |
| Periprocedural antiplatelets | 83 (94.3) | 52 (91.2) | 31 (100.0) | 0 |
| Number of periprocedural antiplatelets |  |  |  | 4.6 |
| 1 | 41 (47.1) | 25 (44.6) | 16 (51.6) |  |
| 2 | 33 (37.9) | 19 (33.9) | 14 (45.2) |  |
| 3 | 9 (10.3) | 8 (14.3) | 1 (3.2) |  |
| *Missing/Not applicable* | 4 (4.6) | 4 (7.1) | 0 (0.0) |  |
| Periprocedural antiplatelet 1 |  |  |  | 9.2 |
| Aspirin | 46 (52.9) | 23 (41.1) | 23 (74.2) |  |
| Cangrelor | 6 (6.9) | 6 (10.7) | 0 (0.0) |  |
| Eptifibatid/Integrilinn | 4 (4.6) | 3 (5.4) | 1 (3.2) |  |
| Ticagrelor | 1 (1.1) | 1 (1.8) | 0 (0.0) |  |
| Tirofiban | 22 (25.3) | 16 (28.6) | 6 (19.4) |  |
| *Missing/Not applicable* | 8 (9.2) | 7 (12.5) | 1 (3.2) |  |
| Periprocedural antiplatelet 2 |  |  |  | 59.8 |
| Abciximab | 1 (1.1) | 1 (1.8) | 0 (0.0) |  |
| Aspirin | 2 (2.3) | 2 (3.6) | 0 (0.0) |  |
| Cangrelor | 3 (3.4) | 1 (1.8) | 2 (6.5) |  |
| Clopidogrel | 6 (6.9) | 4 (7.1) | 2 (6.5) |  |
| Eptifibatid/Integrilinn | 2 (2.3) | 2 (3.6) | 0 (0.0) |  |
| Ticagrelor | 1 (1.1) | 0 (0.0) | 1 (3.2) |  |
| Tirofiban | 20 (23.0) | 11 (19.6) | 9 (29.0) |  |
| *Missing/Not applicable* | 52 (59.8) | 35 (62.5) | 17 (54.8) |  |
| Periprocedural antiplatelet 3 |  |  |  | 78.2 |
| Clopidogrel | 1 (1.1) | 1 (1.8) | 0 (0.0) |  |
| Eptifibatid/Integrilinn | 14 (16.1) | 7 (12.5) | 7 (22.6) |  |
| Prasugrel | 1 (1.1) | 1 (1.8) | 0 (0.0) |  |
| Ticagrelor | 2 (2.3) | 2 (3.6) | 0 (0.0) |  |
| Tirofiban | 1 (1.1) | 1 (1.8) | 0 (0.0) |  |
| *Missing/Not applicable* | 68 (78.2) | 44 (78.6) | 24 (77.4) |  |
| Postprecedural antiplatelets | 81 (93.1) | 52 (92.9) | 29 (93.5) | 1.1 |
| Number of periprocedural antiplatelets |  |  |  | 5.7 |
| 1 | 14 (16.1) | 9 (16.1) | 5 (16.1) |  |
| 2 | 68 (78.2) | 44 (78.6) | 24 (77.4) |  |
| *Missing/Not applicable* | 5 (5.7) | 3 (5.4) | 2 (6.5) |  |
| Postprocedural antiplatelet 1 |  |  |  | 9.2 |
| Aspirin | 64 (73.6) | 39 (69.6) | 25 (80.6) |  |
| Clopidogrel | 6 (6.9) | 4 (7.1) | 2 (6.5) |  |
| Ticagrelor | 8 (9.2) | 6 (10.7) | 2 (6.5) |  |
| Tirofiban | 1 (1.1) | 1 (1.8) | 0 (0.0) |  |
| *Missing/Not applicable* | 8 (9.2) | 6 (10.7) | 2 (6.5) |  |
| Postprocedural antiplatelet 2 |  |  |  | 19.5 |
| Aspirin | 14 (16.1) | 10 (17.9) | 4 (12.9) |  |
| Clopidogrel | 42 (48.3) | 27 (48.2) | 15 (48.4) |  |
| Prasugrel | 3 (3.4) | 1 (1.8) | 2 (6.5) |  |
| Ticagrelor | 11 (12.6) | 8 (14.3) | 3 (9.7) |  |
| *Missing/Not applicable* | 17 (19.5) | 10 (17.9) | 7 (22.6) |  |

| **Supplement Table 2: Outcomes LVO vs MDVO** | | | |
| --- | --- | --- | --- |
|  | LVO | MDVO | p-value |
| n | 173 | 87 |  |
| mRS 90 days (median) | 3 [1, 6] | 3 [2, 5] | 0.509 |
| mRS 90 days (levels) |  |  |  |
| 0 | 26 (15.0) | 10 (11.5) |  |
| 1 | 26 (15.0) | 10 (11.5) |  |
| 2 | 22 (12.7) | 10 (11.5) |  |
| 3 | 22 (12.7) | 14 (16.1) |  |
| 4 | 19 (11.0) | 17 (19.5) |  |
| 5 | 13 (7.5) | 5 (5.7) |  |
| 6 | 45 (26.0) | 21 (24.1) |  |
| Functional independence 90 days | 74 (42.8) | 30 (34.5) | 0.249 |
| sICH | 9 (5.3) | 7 (8.4) | 0.491 |
| NIHSS 24 hours | 8.0 [3.0, 18.0] | 9.0 [4.0, 17.0] | 0.718 |
| NIHSS change | -2.0 [-6.0, 4.0] | 0.0 [-5.0, 4.8] | 0.256 |
| NIHSS percentage change | -0.2 [-0.6, 0.4] | 0.0 [-0.4, 0.6] | 0.075 |
| Death | 45 (26.0) | 21 (24.1) | 0.86 |
| Postprocedural stent occlusion | 17 (9.8) | 12 (13.8) | 0.542 |
|  |  |  |  |

* The table shows that the outcomes do not differ between the matched groups, meaning that the outcomes of patients with MDVOs are not different from matched patients with LVOs.

** * P-values represent the unadjusted comparison between both groups using an ordinal regression model for mRS at 90 days, Wilcoxon signed rank tests for the outcomes related to NIHSS, and a Chi-squared or Fisher exact test for the binary outcomes.
